# Impact of CoAP and MQTT on NB-IoT System Performance †

**DOI:** 10.3390/s19010007

**Published:** 2018-12-20

**Authors:** Anna Larmo, Antti Ratilainen, Juha Saarinen

**Affiliations:** 1 Ericsson Research, 02420 Jorvas, Finland; antti.ratilainen@ericsson.com; 2 Ukkoverkot, 00180 Helsinki, Finland; jsaari@gmail.com

**Keywords:** CoAP, IoT, MQTT, NB-IoT, TCP, UDP

## Abstract

The IoT protocols used for data transfer in the application layer, namely the Constraint Application Protocol (CoAP) and Message Queue Telemetry Transport (MQTT) have dependencies to the transport layer. The choice of transport, Transmission Control Protocol (TCP) or the User Datagram Protocol (UDP), on the other hand, has an impact on the Internet of Things (IoT) application level performance, especially over a wireless medium. Furthermore, we touch upon the impact of different security solutions. The motivation of this work is to look at the impact of the protocol stack on performance over a narrowband IoT (NB-IoT) link. The use case studied is infrequent small reports sent from the sensor device to a central cloud storage over a last mile radio access link. We find that while CoAP/UDP based transport performs consistently better both in terms of latency, coverage, and system capacity, MQTT/TCP also works when the system is less loaded.

## 1. Introduction

The core idea of the Internet of Things (IoT) is to use the Internet Protocol (IP), mainly IPv6, to communicate between connected devices and services in the cloud. A plethora of standards addressing the IoT on different levels of the protocol stack are available today and may be combined in different ways.

The IoT umbrella covers many use cases. Here we focus on use cases where small data transmissions are expected from and to sensors and actuators. Such use cases are for example connected lighting, connected home sensors and actuators, smart metering, smart agriculture, etc.

Most of the popular IoT applications today use either the Internet Engineering Task Force (IETF) standardized Constraint Application Protocol (CoAP) or the OASIS specified Message Queue Telemetry Transport (MQTT) as the data transfer of choice. Other examples of IoT application layer transfer protocols are the Hyper Text Transfer Protocol, Data Distribution Service and Advanced Message Queuing Protocol [1].

The requirements of the use case set boundary conditions to the reliability and latency of the communication link. If the use case involves collecting large amounts of sensor data for statistical purposes, and the traffic is mostly from the sensor to the cloud, it may be acceptable to lose occasional packets. Even in such scenarios, however, reliable transport may be required on occasion when firmware updates or security patches are done on devices.

The physical layer and the medium access control, i.e., L1 and L2, may differ greatly depending what kind of access technology is used. In the end to end path of an IoT application data flow, there may be fast fiber links, Ethernet cabling, as well as radio access links over a cellular network. In this paper, we focus on the low power wide area technologies and especially to cellular network links realized with the narrowband IoT (NB-IoT) technology. NB-IoT is one of the cornerstone massive IoT technologies forming the baseline for 5G. As part of the 5G technology family, NB-IoT systems always have support for IP based traffic.

Other low power wide area technologies are, for example Sigfox and LoRa. Both Sigfox and LoRa differ from NB-IoT in that they run over the unlicensed spectrum while NB-IoT runs over licensed spectrum. Both technologies have an optimized last hop where the use of TCP, or even IP, is not common. This has been identified by the Internet Engineering Task Force (IETF) where work is currently ongoing to define header compression to enable better IP support [2]. 

In this work, we study how the combination of different higher layer stacks impact the NB-IoT system performance with the hypotheses that CoAP will perform better than MQTT.

### 1.1. Previous Work

Previous work on comparing CoAP and MQTT has been done in the form of small scale trials over an unnamed radio technology [3] and by analytical means [4]. In [3], a healthcare use case was realized with prototype hardware. The performance of the two protocols was then measured when a set of medical sensor data was transferred over an unspecified wireless link. It was found that unpredictable packet loss is handled poorly by CoAP but better by MQTT due to use of TCP. In [4], the performance of the two protocols was studied over very lossy links. It was found that by tuning CoAP parameters, the performance can be greatly improved and CoAP often performs better than MQTT due to the lower overhead, although some level of packet loss needs to be accepted when UDP is used. 

The applicability of TCP (which MQTT uses for transport) for IoT has been analyzed in [5]. The analysis show that traditional TCP has several significant drawbacks for IoT traffic, such as increased overhead and lack of flexibility for loss-tolerant applications. A more lightweight implementation TCP is introduced. 

In addition, the two lightweight protocols have been compared in [6], where MQTT and CoAP are adopted in smartphone applications. The study shows that CoAP can be a valid alternative to MQTT in certain scenarios. However, smartphone radio connectivity is much less demanding and doesn’t introduce similar challenges as NB-IoT in terms of throughput, availability, coverage and battery lifetime.

Neither of these papers consider in detail the radio layer, and the implications of constrained radio technology on higher layers. Our contribution to the discussion is to study the performance over realistically modeled actual physical layer and medium access control (MAC) protocols and see how the combination of the protocols all the way from the application layer down to the physical layer impacts the application performance.

We studied the performance of CoAP and MQTT over two different last hop wireless links, Bluetooth and Wi-Fi in [7]. These links can be considered to be not narrow band, as the bandwidths are larger than in NB-IoT. The smartphone links studied in [6] are also not narrow band. To best of our knowledge, application level performance has not been studied over NB-IoT before. In [8], a NB-IoT technology overview and full stack was experimented in a cloud-RAN implementation but impact of higher layer on performance was not studied. 

### 1.2. IoT Stack for Massive MTC

**Figure 1:**
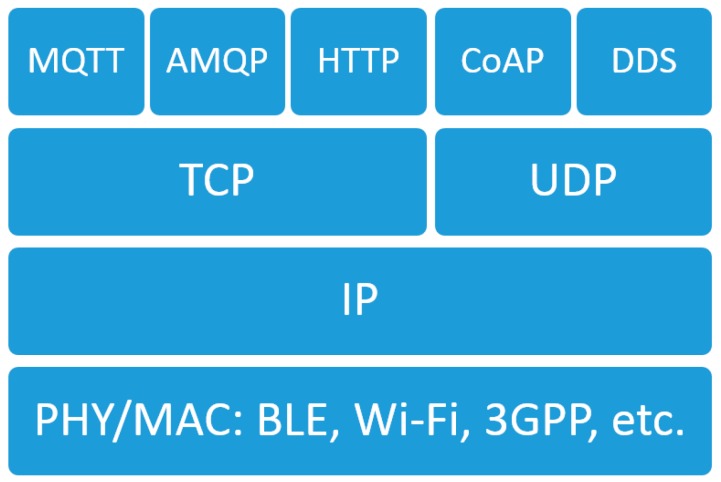
Different options for the IoT stack.

Several realizations of the full IoT stack have been discussed in the industry and academia. However, it is likely that convergence to a single option will not happen in the near future due to different characteristics of the large variety of use cases grouped under IoT. Figure 1 depicts different options for the IoT stack on different layers for the massive Machine Type Communication (MTC) use cases.

When CoAP [9] is used in the transfer layer, UDP is the default transport assumed. It should be noted though that in the IETF, TCP transport for CoAP is currently discussed [10]. MQTT [11], relies on TCP on the transport layer, while a version for UDP called MQTT-SN has been discussed as well. More broadly, Data Distribution Service for Real-Time Systems typically utilizes UDP, while with Hypertext Transfer Protocol and Advanced Message Queuing Protocol, TCP is used instead by default. To understand how different stacks may impact application level performance and system design, the differences between the two different transports need to be understood.

The key differences between TCP and UDP are that UDP is connectionless, light weight, and unreliable, whereas TCP brings more overhead but also reliability with its connection oriented confirmed delivery of packets. A TCP connection requires a three-way handshake between the sensor and the cloud end at the setup phase. On the contrary, a UDP data transmission does not require any setup or bi-directionality; the packet is simply sent from the transmitter end to the receiver end at a suitable time. TCP fast open is a variant of the TCP protocol, which allows data transmission along with TCP SYN and SYN-ACK transmissions [12]. Although TCP fast open will simplify TCP usage for IoT, we have chosen to limit our study on the legacy TCP as it still is widely used.

Security is a key part of any IoT stack. There are different ways to provide security and typical options for the discussed stack are to use either Transport Layer Security (TLS) for TCP or Datagram Transport Layer Security (DTLS) for UDP. Security solutions typically bring overhead and may introduce extra handshakes to the procedure as well. However, in this work we assume that the security handshakes have already been performed at an earlier time, e.g., out-of-bound at deployment or manufacturing phase of the sensor life cycle.

CoAP is a RESTful application layer transfer protocol specified by the IETF [9]. CoAP typically is run over UDP connections, although work to adapt to TCP is also ongoing [10]. CoAP may be run in two different modes, namely the confirmed delivery mode and the non-confirmed delivery mode. For sensor reporting with relaxed reliability requirement the non-confirmed mode is enough. Confirmations may be also requested only for some packets. CoAP headers are four Bytes long as a minimum. Optional fields may increase header size when needed. 

MQTT [11] is an application layer publish/subscribe transfer protocol specified by OASIS. In MQTT, topics are set up between the client and the server which are then subscribed and published to. For sensor reporting, the sensor client registers at a server to publish sensor data, and vice versa register for topics to receive updates when needed. MQTT headers are one Byte as a minimum. Optional fields may increase header size when needed.

#### Overview of NB-IoT

Narrowband Internet of things (NB-IoT) is a cellular radio access technology standardized in 3rd generation partnership project (3GPP), and it has been designed to provide low power wide area network (LPWAN) connectivity in licensed spectrum. The design principles for NB-IoT have been long device battery life, low device complexity, support for massive number of devices and support for high coverage to reach devices in wide areas as well as in challenging locations [13]. 

As the name suggests, NB-IoT can be deployed in a narrow bandwidth of 200 kHz and offers coverage up to 164 dB maximum coupling loss (MCL). There are three deployment options for NB-IoT, inside (*in-band*) or in the guard band (*guard-band*) of an LTE carrier, or *stand-alone* mode in its own spectrum. The coverage enhancements are mainly achieved with transmission repetitions together with power boosting.

LTE design has been reused for NB-IoT to a large extent, including numerologies, channel coding and modulation schemes, and higher layer protocols, which allows quick and easy deployment and further development of NB-IoT products within existing LTE networks. In uplink, a new subcarrier spacing of 3.75 kHz is introduced in addition to the 15 kHz used also in the legacy LTE. Resource allocation of using less than twelve 15 kHz subcarriers in the uplink is also possible for NB-IoT. Narrowband physical random-access channel (NPRACH) supports only 3.75 kHz subcarrier spacing, while for narrowband physical uplink shared channel (NPUSCH) 3.75 kHz (in single tone-transmission) and 15 kHz (in both, single-tone and multi-tone transmission) subcarrier spacing are supported.

To reduce device complexity, several simplifications to legacy LTE mechanisms have been made, including supporting only half-duplex frequency division duplex (HD-FDD), and only one receiving antenna, only quadrature phase shift keying (QPSK) and convolutional code is used in the downlink, in the uplink single-tone transmission with π/2-binary phase shift keying (BPSK) and π/4-QPSK are supported. To further reduce complexity, several other relaxations and optimizations e.g. in requirements of acquiring and decoding downlink channels as well as performing uplink transmissions have been introduced. To improve battery life, there have been enhancements to legacy LTE mechanisms and introduction of new mechanisms e.g. extended discontinuous reception (eDRX), power saving mode (PSM, multi-carrier operations have been introduced especially for NB-IoT.

In addition to the brief overview above focusing mainly on Release 13 NB-IoT, NB-IoT has been developed and is being developed further, as 3GPP Release 14 and Release 15 introduced further enhancements for NB-IoT. Currently 3GPP are working on Release 16, which will also introduce further enhancements for NB-IoT. 

## 2. Performance Evaluation

The evaluations discussed in the following were performed using an Ericsson internal event-based radio network system simulator. This used simulator is similar in structure and capabilities to the commonly used NS3 simulator with detailed modeling of application, IP, transport, network and link layers for NB-IoT. In the following we describe the simulator model in detail.

### 2.1. NB-IoT Modeling

The used simulator models NB-IoT protocol stack in high detail, spanning from Radio Resource Control (RRC) protocol layer in the control plane, and Packet Data Convergence Protocol (PDCP) layer in user plane down to MAC and physical layers. This includes detailed modelling of RRC, PDCP, Radio Link Control (RLC) and MAC layers, and implementation of relevant NB-IoT physical channels (NPRACH: Narrowband Physical Downlink Control Channel, NPDCCH; Narrowband Physical Downlink Shared Channel, NPDSCH; NPUSCH). These protocol stacks are implemented both in the base station and the device, and the NPDSCH and NPUSCH physical channel model noise and interference coming from neighbor cells and from the transmissions of other devices, while other physical channels are more simplistic and implement probability-based errors. 

The propagation model for the radio channels follow the 3GPP typical urban channel model [14], where the generated devices are indoors resulting in 20 dB additional attenuation. The NB-IoT carrier in the simulations is deployed in 900 MHz carrier. The maximum transmission power of the base stations is 40 W, and base stations have 2 receive/2 transmissions antennas, while the devices have maximum transmission power of 0.2 W and 1 receive/1 transmission antenna. The scheduling algorithm used by the base stations is round-robin.

Three coverage enhancement (CE) levels are configured with certain repetition configuration on each of the CE-level, with Maximum Coupling Loss (MCL) thresholds determining on which CE-level the device should perform random access and consequently, the data transmission. 

### 2.2. Above IP

TCP model used was tuned for NB-IoT by setting the initial window size at three segments and setting the congestion window size at 10 segments. In order to avoid spurious retransmissions, the initial round trip time estimate was set to one second. 

Simplified CoAP and MQTT models were used with a payload size on top of the transport protocol. Detailed models of IP, TCP and UDP layers were used as well as detailed modelling of radio network protocols, RRC, PDCP, RLC, and MAC layers, as described in the previous section. 

The cloud end point for the data connection is assumed to be in a central location. Simplistic modelling of the Internet is used, which adds 4 ms delay in both uplink and downlink directions.

### 2.3. Traffic Modeling

Sensor reports were randomly generated by means of a Poisson process. For TCP, the session is terminated after each sensor report was received in the other end of the connection, however, the termination procedure of a TCP connection has not been explicitly modelled. Sensors are distributed uniformly around the simulation area. When a new sensor report is to be sent a UE is created to the system to transmit the report. When a UE arrives to the system, it immediately tries to establish a RRC connection and transmit its application layer data. After successful transmission of the data the UE is removed from the system after a short, fixed delay to make sure all data transfer has ended on all layers.

### 2.4. Simulation Scenario

The simulated scenario consists of 7 base stations with 3 sectors i.e. cells each, creating a network of 21 hexagonal cells. UEs are created uniformly across the simulation area, each connecting to the base station which controls that particular cell. 

In the simulations the UEs transmit a packets of size 25 bytes or 400 bytes in uplink, the transfer protocol headers for CoAP or MQTT are assumed to be included in this payload. The arrival rate of UEs to the system is varied to show the impact of system load on the performance.

The arrival rate of 1 MTC request per second per cell corresponds to 21 devices per second in the system, which is around 75,600 devices transmitting data in the system in one hour. A load of 2 MTC requests per second thus corresponds to ~150,000 requests in the system per hour, and a load of 4 MTC requests per second per cell to ~300,000 requests in the system per hour respectively. These loads may seem high as NB-IoT has been design target was to support 50,000 devices per cell [15], however, the objective is to test the limits of the system. 

## 3. Results

Simulations of MTC devices communicating over a CoAP stack with UDP and a MQTT stack with TCP transfer layer over NB-IoT were performed to study the impact the choice of transport layer has on the user and system performance. The main aspects studied are the extra delay and the impact on system load caused by TCP usage when compared to using UDP. We focus on simple sensor report traffic in the uplink direction. When using CoAP, this type of traffic can be sent non-confirmed, while with the MQTT stack TCP will provide reliability. We simulate also the CoAP confirmed case to show the effect using the confirm mode has on the application level performance.

### 3.1. Throughput

Comparing average throughputs over different load points gives an understanding of how the transport layer impacts the performance. The throughput is calculated such that we take the data size of the payload to be transferred and divide that with the delay measurement from the time the packet is generated to the buffer in the device until the time the full packet is received in the cloud end. As the data sizes are too small to reach the full capacity of the link, the larger data size shows better throughput. 

**Figure 2:**
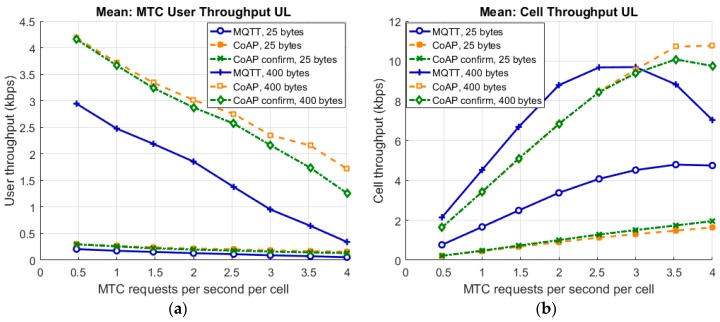
MTC application level throughput as a function of system load for (**a**) a single device; (**b**) on cell level.

Figure 2 shows the mean throughput as a function of system load as experienced by a single device (a) and on the cell level (b). As can be seen in Figure 2a, CoAP shows a higher application layer throughput than MQTT. This is due to the TCP three-way handshake contribution to the delay for the data transfer in the beginning of the communication. CoAP confirm performance degrades faster than the CoAP performance without confirm messages for the larger file as the load increases in the system. This is most likely due to the increased load from CoAP retransmissions that load the system further when the packets get delayed due to congestion. A lower throughput may increase the power consumption at the device as the device needs to remain in connected state for a longer period of time.

Figure 2b shows how the system reaches its saturation point earlier for MQTT. This can be seen as the point where the cell throughput reaches its peak. When the cell throughput starts decreasing under load the air interface is no longer efficiently used. The second curve to start bending downwards is the CoAP confirm case. CoAP with no confirm reaches its saturation point outside of the plot area. 

### 3.2. Service Availability

**Figure 3:**
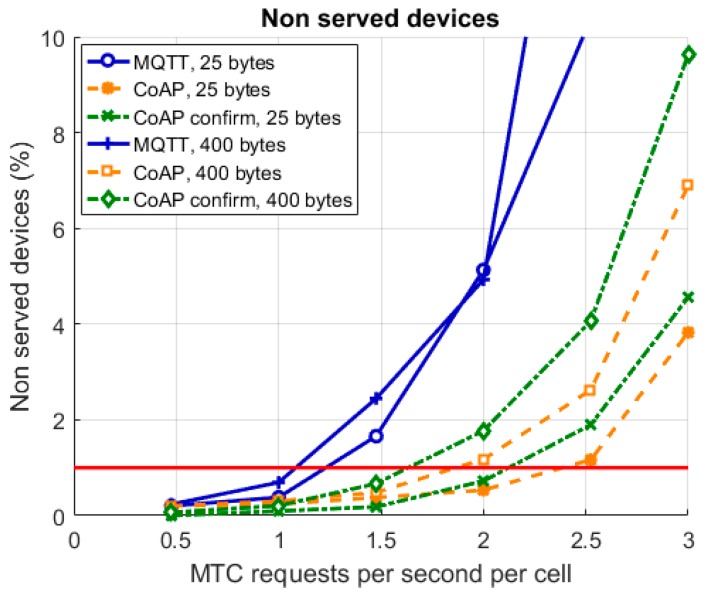
Percentage of non-served devices as a function of load.

Service availability is defined in this study in terms of delivery delay. In case the system has not been able to deliver the data transmission from the device to the cloud in 20 s, the device is considered as non-served. As can be seen in Figure 3, all stack options reach a service availability above 99% in the two lowest load points studied. At load 1.5 MTC requests per cell however, the MQTT service availability start rapidly to decrease reaching 95% at 2 MTC requests per second and cell then further lowering below 90% for the consecutive studied loads. 

CoAP survives the increase of load better but for the larger data size service availability drops below 99% at 2 MTC requests per second and cell. The curves shoot upwards in the order of generated load. Most load is generated with the larger file size over MQTT due to the TCP overhead. Second most load is generated by the smaller file size over MQTT, again due to the TCP overhead. After MQTT come the two plots for CoAP confirm, first larger file size and then smaller file size. Retransmissions cause the extra load. Best service availability is, perhaps counter intuitively, reached when no reliability measures are in effect and the load is thus the lowest. 

### 3.3. Coverage

**Figure 4:**
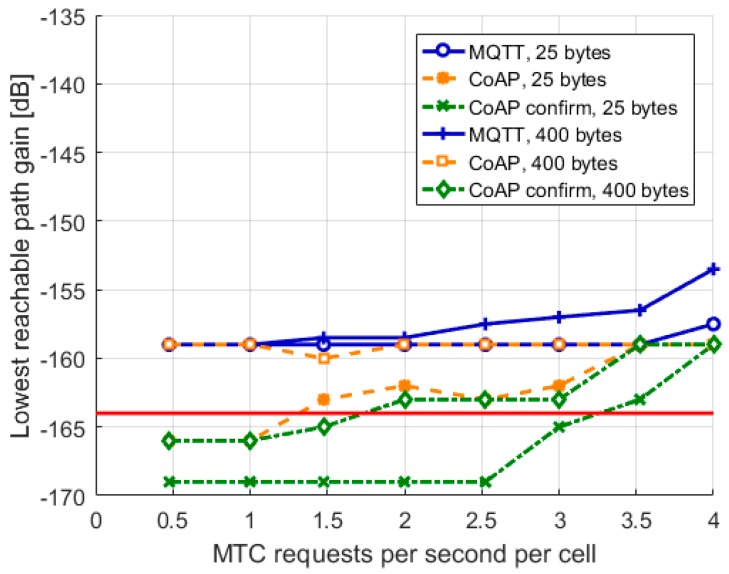
Lowest reachable path gain.

Finally, we look at impact of the stack on coverage. This is depicted in Figure 4, where we show the served devices with the lowest reachable path gain. Two observations can be made from the figure: first, the lowest reachable path gains for MQTT are on average higher than the lowest reachable path gains for CoAP. Also, enabling CoAP confirm helps to reach devices in deep coverage better. This basically hints that CoAP service has a better coverage than MQTT service, especially when confirm is used. Second, as load increases, the lowest reachable path gain also increases. It seems that as the increased number of devices in the system demand more system resources, the devices in deep coverage are left without. Thus, it seems that in high load, the system is not able to allocate enough resources for users in bad coverage.

## 4. Discussion

In this work, we studied 5G massive IoT realization over a NB-IoT system. The simulation study performed confirms the working hypotheses that MQTT as a TCP based system impacts negatively both the device perceived throughput and the system load as well as service availability and coverage. When confirmation of sensor report transmission is required, CoAP confirm provides a lightweight and cheap alternative to TCP. In fact, adding reliability to the transport layer in terms of TCP causes a lower service availability than using a simple UDP transport as the increased load caused by the protocol overhead saturates the system sooner. 

Additional overhead from TCP retransmissions also impacts service coverage negatively. However, in this case, it seems that the additional overhead from CoAP confirm helps to reach devices in deep coverage better compared to no retransmission mode.
